# *trans*-Bis(1-cyclo­hexyl­pyrrolidin-2-one)dinitratopalladium(II)

**DOI:** 10.1107/S1600536809045930

**Published:** 2009-11-07

**Authors:** Yuya Takahashi, Yasuhisa Ikeda

**Affiliations:** a Research Laboratory for Nuclear Reactors, Tokyo Institute of Technology, 2-12-1-N1-34 Ookayama, Meguro-ku, Tokyo 152-8550, Japan

## Abstract

In the title compound, [Pd(NO_3_)_2_(C_10_H_17_NO)_2_], the Pd^II^ centre is located on an inversion center and is coordinated in a square-planar geometry by two O atoms of the monodentate nitrate groups and two carbonyl O atoms of the 1-cyclo­hexyl­pyrrolidin-2-one ligands.

## Related literature

For general background to ambidentate ligands, see: Fairlie & Taube (1985 ▶); Rack *et al.* (2003 ▶); Sigel & Martin (1982 ▶). For amide complexes of metal ions, see: Anget *et al.* (1990 ▶); Curtis *et al.* (1983 ▶). Pankratov *et al.* (2004 ▶); Wayland & Schramm (1969 ▶); Rheingold & Staley (1988 ▶). For the structures of ambidentate ligand complexes of Pd^II^, see: Johnson *et al.* (1981 ▶); Johansson *et al.* (2001 ▶); Langs *et al.* (1967 ▶). For the structures of nitrate complexes of Pd^II^, see: Bennett *et al.* (1967 ▶); Adrian *et al.* (2006 ▶); Rath *et al.* (1999 ▶); Bray *et al.* (2005 ▶); Cerdà *et al.* (2006 ▶); Gromilov *et al.* (2008 ▶); Khranenko *et al.* (2007 ▶); Laligant *et al.* (1991 ▶). For a discussion on the relationship between bond lengths and ligand donicities, see: Gutmann (1967 ▶, 1968 ▶); Koshino *et al.* (2005 ▶).
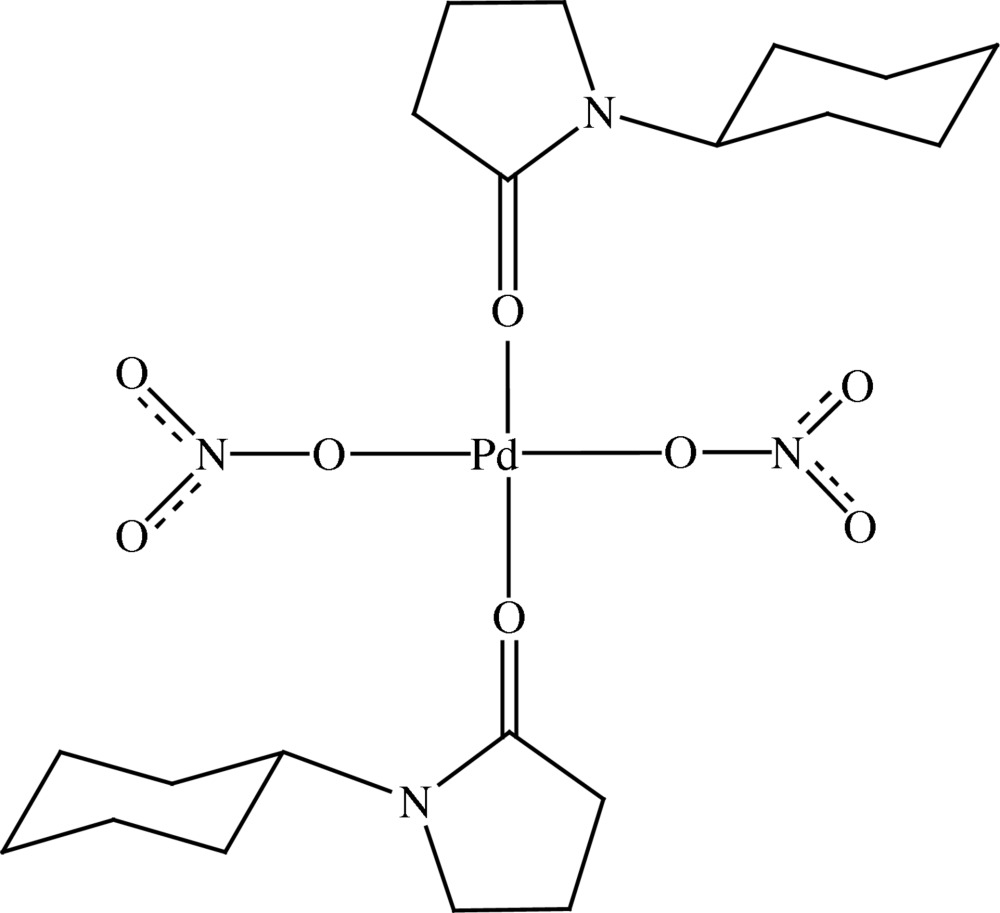


## Experimental

### 

#### Crystal data

[Pd(NO_3_)_2_(C_10_H_17_NO)_2_]
*M* *_r_* = 564.91
Triclinic, 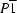

*a* = 7.6431 (5) Å
*b* = 9.8892 (8) Å
*c* = 10.1118 (7) Å
α = 60.8650 (19)°
β = 66.057 (2)°
γ = 68.845 (2)°
*V* = 597.24 (7) Å^3^
*Z* = 1
Mo *K*α radiation
μ = 0.83 mm^−1^
*T* = 173 K
0.78 × 0.41 × 0.07 mm

#### Data collection

Rigaku R-AXIS RAPID diffractometer
Absorption correction: numerical (*ABSCOR*; Higashi, 1999 ▶) *T*_min_ = 0.754, *T*_max_ = 0.943
5836 measured reflections
2696 independent reflections
2662 reflections with *I* > 2σ(*I*)
*R*_int_ = 0.021

#### Refinement

*R*[*F* ^2^ > 2σ(*F* ^2^)] = 0.020
*wR*(*F* ^2^) = 0.052
*S* = 1.07
2696 reflections
152 parameters
H-atom parameters constrained
Δρ_max_ = 0.32 e Å^−3^
Δρ_min_ = −0.93 e Å^−3^

### 

Data collection: *PROCESS-AUTO* (Rigaku, 1998 ▶); cell refinement: *PROCESS-AUTO*; data reduction: *CrystalStructure* (Rigaku/MSC, 2006 ▶); program(s) used to solve structure: *SIR92* (Altomare *et al.*, 1994 ▶) and *DIRDIF99* (Beurskens *et al.*, 1999 ▶); program(s) used to refine structure: *SHELXL97* (Sheldrick, 2008 ▶); molecular graphics: *ORTEP-3* (Farrugia, 1997 ▶); software used to prepare material for publication: *CrystalStructure*.

## Supplementary Material

Crystal structure: contains datablocks global, I. DOI: 10.1107/S1600536809045930/br2124sup1.cif

Structure factors: contains datablocks I. DOI: 10.1107/S1600536809045930/br2124Isup2.hkl

Additional supplementary materials:  crystallographic information; 3D view; checkCIF report

## Figures and Tables

**Table 1:** Selected bond lengths (Å)

Pd(1)—O(1)	2.0092 (11)
Pd(1)—O(2)	2.0112 (15)

**Fig. 1.**
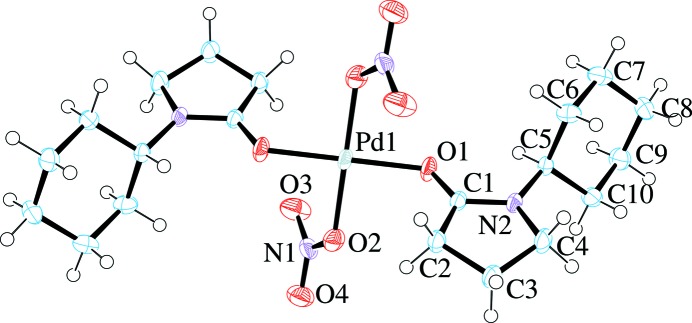
The ORTEP view of trans-Pd(NO3)2(NCP)2 complex with the atomic numbering. The thermal ellipsoids are drawn at 50% probability.

**Fig. 2.**
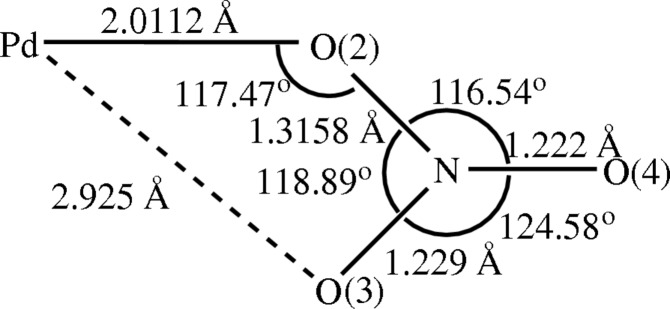
The configuration of coordinated nitrate in trans-Pd(NO3)2(NCP)2.

**Fig. 3.**
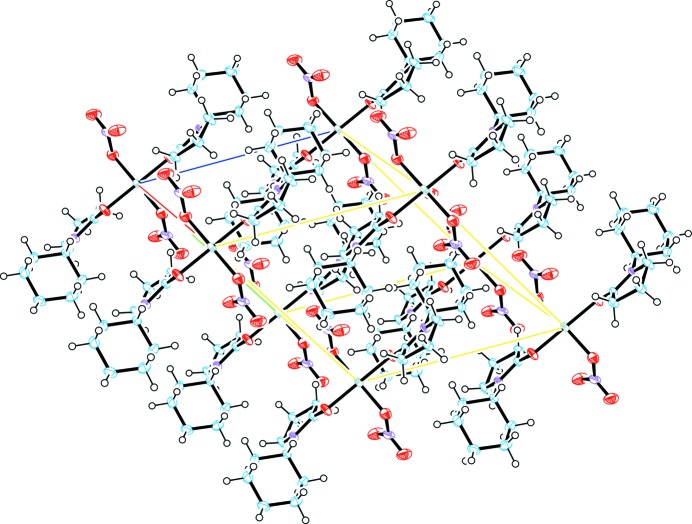
The packing view of trans-Pd(NO3)2(NCP)2 complex. The thermal ellipsoids are drawn at 50% probability.

## supplementary crystallographic information

### Comment

Ambidentate ligands are known as ligands with two different coordination cites, such as thiocyanate ion (N and S), cyanate ion (N and O), dimethyl sulfoxide (DMSO, O and S), and *N,N*-dimethylformamide (DMF, N and O) (Fairlie *et al.*, 1985; Rack *et al.*, 2003; Sigel *et al.*, 1982). For amide complexes of metal ions classified as hard Lewis acids, such as [*M*(NH_3_)_5_(amide)]^3+^ (*M* = Co, Cr) (Anget *et al.*, 1990; Curtis *et al.*, 1983), the O-bonded form is thermodynamically and kinetically more favored than the N-bonded form. On the other hand, Pd^II^ classified as a soft Lewis acid usually exhibits a weak affinity to O-donor ligands. Hence, amide compounds should coordinate to Pd^II^ through a nitrogen atom more preferably. In fact, it has been known that the Pd^II^ complex with 2-pyrrolidone is *N*-bonded form, *i.e.*, *cis*-PdCl_2_(pyrroline-2-ol)_2_ (Pankratov *et al.*, 2004). However, Pd^II^ complexes with O-bonded amides have been also reported, *e.g.*, PdCl_2_(*L*)_2_, Pd(*L*)_4_.(ClO_4_)_2_ (*L* = DMF, *N,N*-dimethylacetamide, *N*-methyl-acetamide, and *N*-methylformamide) (Wayland *et al.*, 1969), and Pd(DMF)_2_(*o*-(*N*-methylliminomethyl)phenyl).BF_4_ (Rheingold *et al.*, 1988). In a similar manner to amides, Pd^II^ complexes with S– and O-bonded DMSO have been reported, such as *trans*-PdCl_2_(DMSO)_2_ with two S-bonded DMSO, and Pd(DMSO)_4_(BF_4_)_2_.DMSO with two S– and O-bonded DMSO and a solvated DMSO (Johansson *et al.*, 1981; Johansson *et al.*, 2001; Langs *et al.*, 1967). In Pd^II^ nitrate complexes, some crystal structures with S–, P–, N–, or O-donor ligand have been reported, *e.g.*, *cis*-Pd(NO_3_)_2_(DMSO)_2_, (Bennett *et al.*, 1967) Pd(NO_3_)_2_(dppm).3CDCl_3_ (dppm = bis(diphenylphosphino)methane), (Adrian *et al.*, 2006; Rath *et al.*, 1999) enPd(NO_3_)_2_ (en = ethylenediamine), (Bray *et al.*,2005; Cerdà *et al.*, 2006) and *trans*-Pd(NO_3_)_2_(H_2_O)_2_ (Gromilov *et al.*, 2008; Khranenko *et al., *2007; Laligant *et al.*, 1991). In all of these complexes, nitrate coordinates to Pd^II^ as the oxygen donor unidentate ligand. So far as we know, *trans*-Pd(NO_3_)_2_(*L*)_2_ (*L*: oxygen donor unidenntate ligand) is only *trans*-Pd(NO_3_)_2_(H_2_O)_2_ and Pd(NO_3_)_2_(O-bonded amide)_2_ has not been reported. We prepared Pd^II^ nitrate complex with the O-bonded amide, *trans*-Pd(NO_3_)_2_(NCP)_2_ (NCP = *N*-cycrohexyl-2-pyrrolidone), and analyzed its crystal structure using the single-crystal X-ray analytical method. An *ORTEP* view of *trans*-Pd(NO_3_)_2_(NCP)_2_ is shown in Fig. 1. In this complex, the configuration around Pd atom is square planar. The nitrate and NCP coordinate to Pd^II^ through their oxygen atoms. The cyclohexyl group of NCP is torsional to pyrrolidone ring. Fig. 2 shows the configuration of coordinated nitrate in *trans*-Pd(NO_3_)_2_(NCP)_2_. From this figure, it is found that the nitrate is planar with O—N—O angles close to 120°, and that the distance of Pd···O(3)is longer than Pd···O(2), and almost same as that of *trans*-Pd(NO_3_)_2_(H_2_O)_2_ (2.926 Å; Khranenko *et al.* (2007). This reflects the fact that in the *trans*-Pd(NO_3_)_2_(NCP)_2_ complex nitrate coordinates to Pd^II^ as the unidentate ligand. As mentioned above, the skeletal structure of *trans*-Pd(NO_3_)_2_(NCP)_2_ is almost same as that of *trans*-Pd(NO_3_)_2_(H_2_O)_2_. However, the Pd—O(NO_3_) distance in *trans*-Pd(NO_3_)_2_(NCP)_2_ is slightly longer than that in *trans*-Pd(NO_3_)_2_(H_2_O)_2_ (1.999 (5) Å) (Khranenko *et al.*(2007)), and the Pd—O(NCP) distance is 0.02 Å shorter than the Pd—O(water) distance (2.030 (5) Å). The differences in Pd—O(*L*) (*L* = H_2_O or NCP) distances are considered to be due to those in electron donicity of *L*, that is, the donor number (28.6) of NCP is larger than that (18.0) of water (Gutmann, 1967, Gutmann,1968, Koshino *et al.*, 2005). Thus, the NCP molecules should more strongly coordinate to the Pd(NO_3_)_2_ moiety than water. This may result in a slightly longer distance of Pd—O(NO_3_) in *trans*-Pd(NO_3_)_2_(NCP)_2_ than in *trans*-Pd(NO_3_)_2_(H_2_O)_2_. Infrared spectrum of *trans*-Pd(NO_3_)_2_(NCP)_2_ in the solid state was measured as a CaF_2_ pellet by Shimadzu FT—IR-8400S spectrophotometer. The carbonyl stretching band of NCP was observed at 1593 cm^-1^, which is lower frequency than that (1670 cm^-1^) of free NCP. The lower shift value (Δ ν = 77 cm^-1^) is comparable to those (68–107 cm^-1^) for other Pd^II^ amide complexes(Wayland *et al.*, 1969). This supports the result of single-crystal analysis that NCP coordinates to the Pd(NO_3_)_2_ moiety through carbonyl oxygen atom. ^1^H and ^13^C NMR spectra of solution prepared by dissolving *trans*-Pd(NO_3_)_2_(NCP)_2_ and (CH_3_)_4_Si into CDCl_3_ were also measured using Jeol ECX-400 NMR spectrometer (^1^H: 399.8 MHz). The ^1^H and ^13^C NMR signals corresponding to free NCP were not observed. Most of ^1^H and ^13^C NMR signals due to coordinated NCP were found to be shifted to lower field compared with those of free NCP. In ^1^H NMR spectrum, the signals of methyne (CH) proton in cyclohexyl group and the methylene protons (N—CH_2_) in pyrrolidone ring were observed as a broad multiplet at 3.78 p.p.m. (0.14 p.p.m. high field shift compared with that of free NCP) and triplet at 3.58 and 3.50 p.p.m. (0.02 and 0.10 p.p.m. low field shift compared with those of free NCP), respectively. In the ^13^C NMR spectrum, carbonyl carbon and methylene carbon (N—CH_2_) in pyrrolidone ring were observed at 180.49 p.p.m. (6.44 p.p.m. low field shift compared with that of free NCP) and 46.09 p.p.m. (3.22 p.p.m. low field shift compared with that of free NCP). These results suggest that even in CDCl_3_ solution two NCP molecules coordinate to Pd^II^. It is worth noting that in spite of the soft Lewis acid all coordination sites of Pd^II^ are occupied by oxygen donor ligands. The present result should be first example for the crystal analysis of *trans*-Pd(NO_3_)_2_(*L*)_2_ complex, in which *L* is the ambidentate ligand with O– and N-bonding sites.

### Experimental

The crystal of *trans*-Pd(NO_3_)_2_(NCP)_2_ was prepared by adding Pd(NO_3_)_2_.2H_2_O (0.8218 g, 3.084 mmol, Kojima Chemicals Co., Inc., 38.85wt% in Pd) to CH_2_Cl_2_ solution of NCP (1.035 g, 6.186 mmol, Aldrich, 99%). The mixture was refluxed for 30 min with stirring and filtered off any undissolved Pd^II^ nitrate. The resulting solution was concentrated to approximately 5 ml, and then diethyl ether was added to form bilayer and to precipitate the complexes. Brown crystals were formed (yield 1.065 g, 59%). Elemental analyses were carried out by LECO CHNS-932 elemental analyzer. Cacl. for H_34_C_20_N_4_O_8_Pd: C, 42.52; H, 6.07; N, 9.92. Found: C, 42.25; H, 5.80; N, 9.88%.

### Refinement

The H atoms of methylene and methyne were placed in calculated positions with C—H = 0.99 and 1.00, respectively. All H atoms were refined as riding on their parent atoms with *U_iso_*(H) = 1.2*U_eq_*(C)

### Crystal data

**Table:**

[Pd(NO_3_)_2_(C_10_H_17_NO)_2_]	*Z* = 1
*M**_r_* = 564.91	*F*(000) = 292.00
Triclinic, *P*1	*D*_x_ = 1.571 Mg m^−^^3^
Hall symbol: -P 1	Mo *K*α radiation, λ = 0.71075 Å
*a* = 7.6431 (5) Å	Cell parameters from 5845 reflections
*b* = 9.8892 (8) Å	θ = 3.3–27.5°
*c* = 10.1118 (7) Å	µ = 0.83 mm^−^^1^
α = 60.8650 (19)°	*T* = 173 K
β = 66.057 (2)°	Platelet, brown
γ = 68.845 (2)°	0.78 × 0.41 × 0.07 mm
*V* = 597.24 (7) Å^3^	

### Data collection

**Table:**

Rigaku R-AXIS RAPID diffractometer	2662 reflections with *F*^2^ > 2σ(*F*^2^)
Detector resolution: 10.00 pixels mm^-1^	*R*_int_ = 0.021
ω scans	θ_max_ = 27.5°
Absorption correction: numerical (*ABSCOR*; Higashi, 1999)	*h* = −9→9
*T*_min_ = 0.754, *T*_max_ = 0.943	*k* = −12→12
5836 measured reflections	*l* = −11→13
2696 independent reflections	

### Refinement

**Table:**

Refinement on *F*^2^	Secondary atom site location: difference Fourier map
*R*[*F*^2^ > 2σ(*F*^2^)] = 0.020	Hydrogen site location: inferred from neighbouring sites
*wR*(*F*^2^) = 0.052	H-atom parameters constrained
*S* = 1.07	*w* = 1/[σ^2^(*F*_o_^2^) + (0.0294*P*)^2^ + 0.1351*P*] where *P* = (*F*_o_^2^ + 2*F*_c_^2^)/3
2696 reflections	(Δ/σ)_max_ = 0.001
152 parameters	Δρ_max_ = 0.32 e Å^−^^3^
Primary atom site location: structure-invariant direct methods	Δρ_min_ = −0.93 e Å^−^^3^

### Fractional atomic coordinates and isotropic or equivalent isotropic displacement parameters (Å^2^)

**Table:**

	*x*	*y*	*z*	*U*_iso_*/*U*_eq_	
Pd(1)	1.0000	1.0000	0.0000	0.02155 (5)	
O(1)	0.92669 (15)	0.93354 (13)	0.23446 (12)	0.0268 (2)	
O(2)	1.18410 (17)	1.12278 (13)	−0.03171 (13)	0.0308 (2)	
O(3)	0.98979 (19)	1.33981 (14)	−0.14453 (18)	0.0454 (3)	
O(4)	1.25913 (19)	1.34980 (16)	−0.12849 (17)	0.0447 (3)	
N(1)	1.14168 (19)	1.27768 (16)	−0.10479 (15)	0.0289 (2)	
N(2)	0.99744 (17)	0.78446 (13)	0.46810 (13)	0.0201 (2)	
C(1)	1.04947 (19)	0.84244 (15)	0.31243 (15)	0.0200 (2)	
C(2)	1.2637 (2)	0.78476 (17)	0.24741 (16)	0.0246 (2)	
C(3)	1.3405 (2)	0.70029 (19)	0.39282 (17)	0.0283 (3)	
C(4)	1.1591 (2)	0.6721 (2)	0.53523 (17)	0.0308 (3)	
C(5)	0.79357 (19)	0.80910 (15)	0.56480 (15)	0.0197 (2)	
C(6)	0.7016 (2)	0.67022 (18)	0.61976 (19)	0.0284 (3)	
C(7)	0.4882 (2)	0.6965 (2)	0.7192 (2)	0.0320 (3)	
C(8)	0.4713 (2)	0.7307 (2)	0.85611 (18)	0.0331 (3)	
C(9)	0.5632 (2)	0.8697 (2)	0.79808 (19)	0.0329 (3)	
C(10)	0.7782 (2)	0.8389 (2)	0.70401 (18)	0.0288 (3)	
H(1)	1.4309	0.5987	0.3943	0.034*	
H(2)	1.4106	0.7671	0.3930	0.034*	
H(3)	0.7185	0.9056	0.4966	0.024*	
H(4)	1.1427	0.5617	0.5814	0.037*	
H(5)	1.1670	0.6947	0.6176	0.037*	
H(6)	0.8353	0.9314	0.6650	0.035*	
H(7)	0.8527	0.7458	0.7733	0.035*	
H(8)	0.5543	0.8869	0.8894	0.040*	
H(9)	0.4907	0.9666	0.7304	0.040*	
H(10)	0.3316	0.7544	0.9134	0.040*	
H(11)	0.5371	0.6359	0.9307	0.040*	
H(12)	0.4091	0.7865	0.6515	0.038*	
H(13)	0.4353	0.6011	0.7612	0.038*	
H(14)	1.3286	0.8742	0.1665	0.030*	
H(15)	1.2847	0.7108	0.2003	0.030*	
H(16)	0.7772	0.5718	0.6833	0.034*	
H(17)	0.7068	0.6578	0.5268	0.034*	

### Atomic displacement parameters (Å^2^)

**Table:**

	*U*^11^	*U*^22^	*U*^33^	*U*^12^	*U*^13^	*U*^23^
Pd(1)	0.02369 (9)	0.02268 (9)	0.01332 (8)	−0.00108 (5)	−0.00923 (5)	−0.00290 (6)
O(1)	0.0252 (5)	0.0317 (5)	0.0153 (4)	0.0013 (4)	−0.0097 (3)	−0.0052 (4)
O(2)	0.0337 (5)	0.0282 (5)	0.0285 (5)	−0.0043 (4)	−0.0169 (4)	−0.0047 (4)
O(3)	0.0352 (6)	0.0284 (5)	0.0613 (8)	−0.0068 (4)	−0.0266 (6)	0.0010 (5)
O(4)	0.0404 (7)	0.0437 (7)	0.0463 (7)	−0.0220 (5)	−0.0156 (5)	−0.0031 (6)
N(1)	0.0270 (6)	0.0299 (6)	0.0209 (5)	−0.0104 (4)	−0.0063 (4)	−0.0008 (4)
N(2)	0.0224 (5)	0.0203 (5)	0.0152 (5)	−0.0035 (4)	−0.0084 (4)	−0.0035 (4)
C(1)	0.0242 (6)	0.0195 (5)	0.0162 (5)	−0.0047 (4)	−0.0083 (4)	−0.0049 (4)
C(2)	0.0229 (6)	0.0275 (6)	0.0181 (6)	−0.0022 (5)	−0.0075 (5)	−0.0059 (5)
C(3)	0.0241 (7)	0.0327 (7)	0.0221 (6)	−0.0021 (5)	−0.0110 (5)	−0.0055 (5)
C(4)	0.0262 (7)	0.0367 (7)	0.0188 (6)	−0.0018 (5)	−0.0123 (5)	−0.0017 (5)
C(5)	0.0228 (6)	0.0197 (5)	0.0151 (5)	−0.0050 (4)	−0.0067 (4)	−0.0042 (4)
C(6)	0.0278 (7)	0.0286 (7)	0.0356 (8)	−0.0083 (5)	−0.0092 (5)	−0.0160 (6)
C(7)	0.0262 (7)	0.0341 (7)	0.0388 (8)	−0.0127 (5)	−0.0082 (6)	−0.0132 (6)
C(8)	0.0292 (7)	0.0403 (8)	0.0232 (7)	−0.0150 (6)	−0.0022 (5)	−0.0064 (6)
C(9)	0.0331 (8)	0.0436 (8)	0.0268 (7)	−0.0137 (6)	−0.0004 (6)	−0.0199 (7)
C(10)	0.0311 (7)	0.0397 (8)	0.0242 (7)	−0.0169 (6)	−0.0014 (5)	−0.0174 (6)

### Geometric parameters (Å, °)

**Table:**

Pd(1)—O(1)	2.0092 (11)	C(9)—C(10)	1.533 (2)
Pd(1)—O(1)^i^	2.0092 (11)	C(2)—H(14)	0.990
Pd(1)—O(2)	2.0112 (15)	C(2)—H(15)	0.990
Pd(1)—O(2)^i^	2.0112 (15)	C(3)—H(1)	0.990
O(1)—C(1)	1.2699 (17)	C(3)—H(2)	0.990
O(2)—N(1)	1.3158 (16)	C(4)—H(4)	0.990
O(3)—N(1)	1.229 (2)	C(4)—H(5)	0.990
O(4)—N(1)	1.222 (2)	C(5)—H(3)	1.000
N(2)—C(1)	1.3200 (17)	C(6)—H(16)	0.990
N(2)—C(4)	1.4754 (19)	C(6)—H(17)	0.990
N(2)—C(5)	1.4713 (15)	C(7)—H(12)	0.990
C(1)—C(2)	1.5020 (17)	C(7)—H(13)	0.990
C(2)—C(3)	1.535 (2)	C(8)—H(10)	0.990
C(3)—C(4)	1.5304 (18)	C(8)—H(11)	0.990
C(5)—C(6)	1.525 (2)	C(9)—H(8)	0.990
C(5)—C(10)	1.525 (2)	C(9)—H(9)	0.990
C(6)—C(7)	1.5351 (19)	C(10)—H(6)	0.990
C(7)—C(8)	1.525 (3)	C(10)—H(7)	0.990
C(8)—C(9)	1.518 (3)		
			
O(1)—Pd(1)—O(1)^i^	180.00 (7)	C(4)—C(3)—H(2)	110.7
O(1)—Pd(1)—O(2)	89.93 (5)	H(1)—C(3)—H(2)	108.8
O(1)—Pd(1)—O(2)^i^	90.07 (5)	N(2)—C(4)—H(4)	111.1
O(1)^i^—Pd(1)—O(2)	90.07 (5)	N(2)—C(4)—H(5)	111.1
O(1)^i^—Pd(1)—O(2)^i^	89.93 (5)	C(3)—C(4)—H(4)	111.1
O(2)—Pd(1)—O(2)^i^	180.00 (6)	C(3)—C(4)—H(5)	111.1
Pd(1)—O(1)—C(1)	121.33 (8)	H(4)—C(4)—H(5)	109.0
Pd(1)—O(2)—N(1)	117.47 (11)	N(2)—C(5)—H(3)	107.6
O(2)—N(1)—O(3)	118.89 (17)	C(6)—C(5)—H(3)	107.7
O(2)—N(1)—O(4)	116.54 (14)	C(10)—C(5)—H(3)	107.6
O(3)—N(1)—O(4)	124.58 (13)	C(5)—C(6)—H(16)	109.5
C(1)—N(2)—C(4)	112.87 (10)	C(5)—C(6)—H(17)	109.5
C(1)—N(2)—C(5)	123.33 (12)	C(7)—C(6)—H(16)	109.5
C(4)—N(2)—C(5)	123.11 (10)	C(7)—C(6)—H(17)	109.5
O(1)—C(1)—N(2)	121.72 (11)	H(16)—C(6)—H(17)	108.1
O(1)—C(1)—C(2)	127.06 (11)	C(6)—C(7)—H(12)	109.4
N(2)—C(1)—C(2)	111.21 (11)	C(6)—C(7)—H(13)	109.4
C(1)—C(2)—C(3)	103.45 (11)	C(8)—C(7)—H(12)	109.4
C(2)—C(3)—C(4)	105.44 (12)	C(8)—C(7)—H(13)	109.4
N(2)—C(4)—C(3)	103.45 (10)	H(12)—C(7)—H(13)	108.0
N(2)—C(5)—C(6)	110.78 (12)	C(7)—C(8)—H(10)	109.4
N(2)—C(5)—C(10)	111.59 (14)	C(7)—C(8)—H(11)	109.4
C(6)—C(5)—C(10)	111.32 (11)	C(9)—C(8)—H(10)	109.4
C(5)—C(6)—C(7)	110.86 (14)	C(9)—C(8)—H(11)	109.4
C(6)—C(7)—C(8)	111.34 (17)	H(10)—C(8)—H(11)	108.0
C(7)—C(8)—C(9)	111.23 (12)	C(8)—C(9)—H(8)	109.5
C(8)—C(9)—C(10)	110.64 (16)	C(8)—C(9)—H(9)	109.5
C(5)—C(10)—C(9)	109.88 (17)	C(10)—C(9)—H(8)	109.5
C(1)—C(2)—H(14)	111.1	C(10)—C(9)—H(9)	109.5
C(1)—C(2)—H(15)	111.1	H(8)—C(9)—H(9)	108.1
C(3)—C(2)—H(14)	111.1	C(5)—C(10)—H(6)	109.7
C(3)—C(2)—H(15)	111.1	C(5)—C(10)—H(7)	109.7
H(14)—C(2)—H(15)	109.0	C(9)—C(10)—H(6)	109.7
C(2)—C(3)—H(1)	110.7	C(9)—C(10)—H(7)	109.7
C(2)—C(3)—H(2)	110.7	H(6)—C(10)—H(7)	108.2
C(4)—C(3)—H(1)	110.7		
			
O(1)—Pd(1)—O(2)—N(1)	−113.91 (10)	C(5)—N(2)—C(1)—O(1)	4.9 (2)
O(2)—Pd(1)—O(1)—C(1)	−66.76 (14)	C(5)—N(2)—C(1)—C(2)	−174.43 (15)
O(1)—Pd(1)—O(2)^i^—N(1)^i^	−66.09 (10)	C(4)—N(2)—C(5)—C(6)	−74.5 (2)
O(2)^i^—Pd(1)—O(1)—C(1)	113.24 (14)	C(4)—N(2)—C(5)—C(10)	50.2 (2)
O(1)^i^—Pd(1)—O(2)—N(1)	66.09 (10)	C(5)—N(2)—C(4)—C(3)	−174.89 (17)
O(2)—Pd(1)—O(1)^i^—C(1)^i^	−113.24 (14)	O(1)—C(1)—C(2)—C(3)	172.09 (18)
O(1)^i^—Pd(1)—O(2)^i^—N(1)^i^	113.91 (10)	N(2)—C(1)—C(2)—C(3)	−8.6 (2)
O(2)^i^—Pd(1)—O(1)^i^—C(1)^i^	66.76 (14)	C(1)—C(2)—C(3)—C(4)	16.71 (19)
Pd(1)—O(1)—C(1)—N(2)	−172.54 (13)	C(2)—C(3)—C(4)—N(2)	−18.6 (2)
Pd(1)—O(1)—C(1)—C(2)	6.7 (2)	N(2)—C(5)—C(6)—C(7)	−179.40 (13)
Pd(1)—O(2)—N(1)—O(3)	2.66 (19)	N(2)—C(5)—C(10)—C(9)	178.01 (11)
Pd(1)—O(2)—N(1)—O(4)	−177.21 (12)	C(6)—C(5)—C(10)—C(9)	−57.65 (14)
C(1)—N(2)—C(4)—C(3)	14.4 (2)	C(10)—C(5)—C(6)—C(7)	55.81 (17)
C(4)—N(2)—C(1)—O(1)	175.65 (17)	C(5)—C(6)—C(7)—C(8)	−54.16 (16)
C(4)—N(2)—C(1)—C(2)	−3.7 (2)	C(6)—C(7)—C(8)—C(9)	55.18 (16)
C(1)—N(2)—C(5)—C(6)	95.31 (17)	C(7)—C(8)—C(9)—C(10)	−57.20 (18)
C(1)—N(2)—C(5)—C(10)	−140.05 (16)	C(8)—C(9)—C(10)—C(5)	58.08 (16)

Symmetry codes: (i) −*x*+2, −*y*+2, −*z*.
